# S02-2: Integrating “routine” practice into the monitoring of physical activity promotion for adults and vulnerable groups: lessons learned

**DOI:** 10.1093/eurpub/ckae114.203

**Published:** 2024-09-26

**Authors:** Leonie Birkholz, Karim Abu-Omar, Peter Gelius, Wolfgang Geidl, Klaus Pfeifer, Sven Messing

**Affiliations:** WHO Collaborating Centre for PA and Public Health, Department of Sport Science and Sport, Friedrich-Alexander-Universität Erlangen-Nürnberg FAU Erlangen-Nürnberg, Germany; WHO Collaborating Centre for PA and Public Health, Department of Sport Science and Sport, Friedrich-Alexander-Universität Erlangen-Nürnberg FAU Erlangen-Nürnberg, Germany; Institute of Sport Sciences, Université de Lausanne, Lausanne, Switzerland; WHO Collaborating Centre for PA and Public Health, Department of Sport Science and Sport, Friedrich-Alexander-Universität Erlangen-Nürnberg FAU Erlangen-Nürnberg, Germany; WHO Collaborating Centre for PA and Public Health, Department of Sport Science and Sport, Friedrich-Alexander-Universität Erlangen-Nürnberg FAU Erlangen-Nürnberg, Germany; Institute of Sport Sciences, Université de Lausanne, Lausanne, Switzerland; WHO Collaborating Centre for PA and Public Health, Department of Sport Science and Sport, Friedrich-Alexander-Universität Erlangen-Nürnberg FAU Erlangen-Nürnberg, Germany

## Abstract

**Purpose:**

To support policy development, a number of tools are available to inform policymakers about the current status of PA promotion in a specific country. However, an exchange between policymakers and researchers revealed a major gap on the systematic assessment of “routine practice”, i.e. PA promotion activities already taking place on a large scale and on a regular basis (e.g., programs run by governmental or civil society organizations such as municipalities, schools, sports federations, or cycling federations). This study aims to provide an overview of routine practice for PA promotion in Germany as part of the newly developed TARGET:PA tool.

**Methods:**

A systematic search for routine practice at the national level was conducted for adults and for vulnerable groups (older adults, adults with noncommunicable diseases). Based on a search on Google and websites of relevant stakeholders in the field of PA promotion in Germany, the results were categorized by relevant sectors and settings (e.g., sport, health, transport). In addition, the efficacy, reach, and maintenance of the identified routine practice were examined. Finally, scientists and stakeholders were involved to verify the routine practice for PA promotion.

**Results:**

The systematic approach yielded more than 100 routine practices of PA promotion in Germany. They focused both on behavior change (e.g., “bike to work” campaign, exercise referral schemes) and on structural interventions (e.g., public funding of sports for all). Most were identified for adults, especially for the sport sector. The results informed the development of policy briefs on PA promotion for adults, older adults, and adults with noncommunicable diseases published by the German Federal Ministry of Health.

**Conclusions:**

Routine practice is particularly relevant for policymakers as it already has a high reach, and substantial public health impact might be generated by further optimizing it rather than introducing new measures. Integrating this aspect into the monitoring of PA promotion could be an added value, and the approach has the potential to be adapted to other countries.

